# *Pneumocystis murina *colonization in immunocompetent surfactant protein A deficient mice following environmental exposure

**DOI:** 10.1186/1465-9921-10-10

**Published:** 2009-02-19

**Authors:** Michael J Linke, Alan D Ashbaugh, Jeffery A Demland, Peter D Walzer

**Affiliations:** 1 Research Service, Veterans Affairs Medical Center, Cincinnati, OH, USA; 2 Division of Infectious Diseases, Department of Internal Medicine, College of Medicine, University of Cincinnati, Cincinnati, OH, USA

## Abstract

**Background:**

*Pneumocystis spp*. are opportunistic pathogens that cause pneumonia in immunocompromised humans and animals. *Pneumocystis *colonization has also been detected in immunocompetent hosts and may exacerbate other pulmonary diseases. Surfactant protein A (SP-A) is an innate host defense molecule and plays a role in the host response to *Pneumocystis*.

**Methods:**

To analyze the role of SP-A in protecting the immunocompetent host from *Pneumocystis *colonization, the susceptibility of immunocompetent mice deficient in SP-A (KO) and wild-type (WT) mice to *P. murina *colonization was analyzed by reverse-transcriptase quantitative PCR (qPCR) and serum antibodies were measured by enzyme-linked immunosorbent assay (ELISA).

**Results:**

Detection of *P. murina *specific serum antibodies in immunocompetent WT and KO mice indicated that the both strains of mice had been exposed to *P. murina *within the animal facility. However, P. *murina *mRNA was only detected by qPCR in the lungs of the KO mice. The incidence and level of the mRNA expression peaked at 8–10 weeks and declined to undetectable levels by 16–18 weeks. When the mice were immunosuppressed, *P. murina *cyst forms were also only detected in KO mice. *P. murina *mRNA was detected in *SCID *mice that had been exposed to KO mice, demonstrating that the immunocompetent KO mice are capable of transmitting the infection to immunodeficient mice. The pulmonary cellular response appeared to be responsible for the clearance of the colonization. More CD4+ and CD8+ T-cells were recovered from the lungs of immunocompetent KO mice than from WT mice, and the colonization in KO mice depleted CD4+ cells was not cleared.

**Conclusion:**

These data support an important role for SP-A in protecting the immunocompetent host from *P. murina *colonization, and provide a model to study *Pneumocystis *colonization acquired via environmental exposure in humans. The results also illustrate the difficulties in keeping mice from exposure to *P. murina *even when housed under barrier conditions.

## Background

*Pneumocystis spp*. are ubiquitous fungal opportunistic pulmonary pathogens found, in man as well as in wild, domesticated, and laboratory animals. *Pneumocystis spp*. are host specific and cross infection between hosts has not been identified [1]. In humans, *P. jirovecii *is a significant cause of pneumonia in immunocompromised patients and despite effective treatments, patients with advanced *Pneumocystis *pneumonia (PcP) have poor outcomes with mortality rates as high as 50% [2]. The source of *Pneumocystis *infection in humans and animals remains unknown, but it has been proposed that persons with colonized with *P. jirovecii *may act as a reservoir of infection and as a source of infectious organisms [3,4]. Results from both human and animal studies demonstrate that colonization with *Pneumocystis *is not a rare event and may lead to worsening of other pulmonary conditions [5-9]. *P. jirovecii *colonization has been associated with increasing the severity of other pulmonary conditions such as chronic obstructive disease and chronic bronchitis [10-13]. Instances of *P. murina *colonization in commercial laboratory mouse colonies have been associated with various defects in the host immune response; however, under experimental conditions normal mice may also become infected [5,14]. A high incidence of colonization has been described in numerous strains and colonies of laboratory rats, but no specific risk factors for colonization of rats with *P. carinii *have been identified. *Pneumocystis *colonization has also been reported in a simian immunodeficiency virus infected macaque model of human acquired immunodeficiency syndrome [10]. In humans, cigarette smoking and certain locations of residence demonstrate a positive correlation with the incidence of *P. jirovecii *colonization [7].

SP-A is a member of the collectin family of proteins and a component of the pulmonary innate immune system [15]. It is the most abundant surfactant protein, but SP-A deficient (KO) mice do not display any obvious pulmonary deficiencies under normal conditions [16]. However, KO mice are more susceptible to infection by a variety of pulmonary pathogens and mount hyperinflammatory responses to some of these infections [17]. The antimicrobial properties of SP-A act through several mechanisms that lead to enhanced clearance of pathogens from the lung. Opsonization by SP-A through interaction of its carbohydrate recognition domain with carbohydrates on the surface of pathogens increases the attachment and uptake of the organisms by alveolar macrophages [18,19]. SP-A increases the microbiocidal actions of macrophages through induction of reactive oxygen-nitrogen species and stimulating chemotaxis [20-22]. SP-A also appears to have a direct microbiocidal effect [23]. Binding of SP-A to the surface of some pathogens results in killing that is caused by permeabilization of the cell membranes or walls of the organisms.

Corticosteroid immunosuppressed SP-A KO mice develop higher levels *P. murina *infection than WT mice [24,25]. Immunocompetent and CD4+ T-cell depleted KO mice also display delayed clearance following infection by intratracheal inoculation compared to WT mice [26]. SP-A appears to act directly and indirectly in the host response to *P. murina *infection; opsonization with SP-A enhances the recognition of *P. murina *by mouse alveolar macrophages and KO mice with *P. murina *infection display a more exuberant inflammatory response than infected WT mice [24,26].

The purpose of this study was to demonstrate that SP-A prevents the development of a *P. murina *colonization in immunocompetent mice following exposure to an environmental source of the organism. In most animal studies, *P. murina *infection is established by a rather intense exposure, i.e., housing naïve mice with animals that have PcP, or by intratracheal or intranasal inoculation of a fixed dose of organisms. By contrast, in this study the mice were not experimentally exposed to *P. murina *but acquired the infection through environmental exposure. Environmental exposure involves a non experimental route of infection, in which the mice come in contact with *P. murina *during standard laboratory animal handling and housing conditions. The advantage of this model is that it more closely resembles the natural course of the infection in humans than animal models that involve exposure to large numbers of organisms. Experiments were designed to test the hypothesis that SP-A acts as an innate immune surveillance molecule protecting the immunocompetent host from *Pneumocystis *colonization.

## Materials and methods

### Generation of C3H/HeN SP-A deficient mice (KO)

Black Swiss SP-A KO mice were generated by targeted gene inactivation as previously described [27]. The SP-A null allele of Black Swiss mice was bred into the C3H/HeN background through nine generations using a PCR-based genotyping strategy to track the neomycin locus of the gene-targeting cassette [16,28]. SP-A KO mice lacked detectable SP-A mRNA or protein and were deficient in tubular myelin. No alteration in the other surfactant proteins, SP-B, SP-C or SP-D, or surfactant phospholipid composition was noted in the animals deficient in SP-A [27].

### Animals

All of the C3H/HeN KO and WT mice and C3H/HeN severe combined immunodeficiency (*SCID*) mice used in these studies were bred and housed at the University of Cincinnati (UC) Laboratory Animal Medicine (LAM) facility. Mice used in these studies were housed in a single room within the LAM facility under barrier conditions: in microisolator cages; with autoclaved food and water; and restricted access of personnel. The cages are changed in a biocontainment hood, 2–3 times per week. Sentinel mice from the animal room are tested for a standard panel of mouse pathogens on a quarterly and semi annual basis. Over the past 5 years, none of these pathogens have been detected in the sentinel mice from the animal room used for these studies. All animal studies conformed to NIH, UC, and the Department of Veterans Affairs guidelines.

### Corticosteroid Immunosuppression Regimen

Mice were immunosuppressed by the addition of dexamethasone (4 mg/l) in their drinking water. Ampicillin (0.5 mg/ml) was added to the drinking water to prevent development of secondary bacterial infection.

### Antibody mediated CD4+T cell depletion

Mice were injected i.p. with 100 ug of GK1.5 antibody 3 times 2 days apart for one week and then once a week for 3 weeks [29].

### Transmission of Infection by Direct Exposure ("seeding")

An immunosuppressed KO mouse heavily infected with *P. murina *or an immunocompetent KO mouse colonized with *P. murina *were housed in the same cage with six *SCID *mice for two weeks. A third group of *SCID *mice were not exposed to KO mice. Transmission of Pneumocystis infection by direct exposure to an animal infected with Pneumocystis has been referred to as "seeding" and the infected mice are referred to as "seeds" [30].

### Infection with *P. murina *by intratracheal inoculation

*P. murina *was isolated and processed for inoculation as previously described [31]. The mice were lightly anesthetized and 10^6 ^*P. murina *cyst forms were introduced into the lung through a tube inserted into the trachea.

### Reverse-transcriptase quantitative PCR (qPCR) quantitation of *P. murina *infection levels

Lungs were removed *en bloc *flash frozen in liquid nitrogen, ground into a fine powder and stored at -70°C for subsequent analyses [25]. Approximately 50 mgs of frozen lung tissue was reconstituted in 1.0 ml Trizol^® ^Reagent (Invitrogen, Carlsbad, CA) and total RNA was isolated. The RNA was treated with RNAase free-DNAase and recovered by phenol:chloroform extraction and ethanol precipitation. The RNA was evaluated in a spectrophotometer at 260 λ and 280 λ. cDNA was made from 1 ug of RNA using the SuperScript™ II RNAase H- Reverse Transcriptase (Invitrogen, Carlsbad, CA) according to the manufacturer's directions. Quantitation of the amount *P. murina *large subunit ribosomal RNA gene (mtLSU) message in the samples was performed on the iCycler iQ Real-Time PCR Detection System (BioRad, Hercules, CA) using a previously described TaqMan assay [32]. The threshold cycle for each sample was identified as the point at which the fluorescence generated by degradation of the TaqMan probe increased significantly above the baseline. To convert the threshold cycle data to *P. murina *nuclei, a standard curve was generated using cDNA made from RNA isolated from 10^7 ^*P. murina *nuclei. The level of infection of the samples was then estimated using the standard curve. The efficiency of the standard curve qPCR reactions consistently approached 100%. Detection of *P. murina *mtLSU with this assay has also been shown to correlate with viability of the organisms [33].

To ensure that high quality RNA was isolated from all samples and that cDNA synthesis was successful, a SybrGreen incorporation qPCR assay for the mouse vimentin gene mRNA was performed on all samples. Primers were designed to amplify a 109 base pair product from mouse vimentin mRNA (Vimentin-Forward Primer 5'-GTGCGCCAGCAGTATGAAAG-3', Vimentin-Reverse Primer 5'-GCATCGTTGTTCCGGTTGG-3'). qPCR was performed using Taq DNA polymerase (Promega, Madison, WI), with SybrGreen (Invitrogen, Carlsbad, CA) added to the buffer, in the iCycler iQ Real-Time PCR Detection System (BioRad, Hercules, CA). The following reaction conditions were used: Cycle 1. 95°C for 3:00 minutes; Cycle 2. 95°C for15 sec, 60°C for 30 sec with 40 repeats. The fluorescent signal generated by incorporation of SybrGreen into the double-stranded product was collected at 86°C for 10 sec during each repeat to determine the threshold cycle for each sample. The fidelity of the qPCR reactions was confirmed by analysis of the melt curve of the vimentin qPCR product. A single peak with an approximate melting temperature of 88°C was consistently identified in the reactions. The efficiency of the vimentin qPCR reactions consistently approached 100%.

### Microscopic Enumeration of *P. murina*

As previously described, microscopic quantitation of *P. murina *cyst forms and nuclei was performed following Cresyl-Echt violet (CEV) and Dif Quik staining, respectively [14]. Data were expressed as log_10 _cysts forms or nuclei per lung. The limit of *P. murina *detection by microscopic evaluation is approximately 2.5 × 10^4 ^cyst forms or nuclei per mouse.

### Cloning, expression, and purification of a fragment of the *P. murina *MSG

Oligonucleotides were designed on the basis of the known sequence of the MSG gene of *P. murina *and were used in polymerase chain reaction (PCR) to generate a fragment of the MSG gene spanning a nucleotides 2139–3040 corresponding to amino acids 713–1013. The sequences of the oligonucleotides were 2139-5'-GAACTCAAGGAAATTGTACGGCAG-3'-2163 and 3040-5'-TGTTCCTGGTGTTGATGGTGCT-3'-3061. Genomic DNA was purified from *P. murina *using the Qiagen kit and used as a template for the PCR reactions. The sequence of the PCR products was confirmed, and they were cloned into the pET30 expression vector (Novagen) in the correct reading frame and were expressed in Escherichia coli. The recombinant proteins were expressed in inclusion bodies within E. coli and were purified by standard methods. In brief, bacterial cultures expressing recombinant MSG fragments were harvested by centrifugation, the cell pellet was sonicated and washed 3 times in binding buffer without urea (5 mM imidazole, 0.5 M NaCl, and 20 mM Tris-HCl [pH 7.9]), and the final pellet was dissolved in binding buffer with 6 M urea. The recombinant preparations were purified by affinity chromatography using HISbinding resin (Novagen), with the urea being removed during the wash stages. Eluted proteins were dialyzed overnight against PBS (pH 7.4), were filter sterilized, and were frozen at -70°C. Protein concentration was determined by A280 using a standard curve generated with bovine serum albumin.

### Analysis of *P. murina *specific serum antibodies by ELISA

It is clear that different subclasses of IgG mediate diverse host defense mechanisms such as binding of IgG to the various Fc receptors on effector cells. Therefore, MSG specific IgG1, IgG2a and IgG2b were measured in this experiment. These subclasses were examined because they are considered to be more reactive with protein epitopes that would be present on the recombinant antigens, as compared to IgG3 that is recognized as being more reactive with carbohydrate epitopes.

Duplicate wells of a 96-well plate were coated overnight with recombinant MSG at 4°C. The plates were washed with PBS-0.1% Tween-20 and then blocked with 1% BSA in PBS for 1 hour at room temperature. Following blocking, the sera were incubated in duplicate wells at a 1/100 dilution in PBS for 1 hour at room temperature. Plates were washed and incubated with a 1/1000 dilution of affinity purified goat anti-rat IgG conjugated to horseradish peroxidase (0.1 mg/ml)(Kirkegaard and Perry Laboratories, Gaithersburg, MD) for 1 hour at room temperature. Following washing, ABTS peroxidase substrate (Kirkegaard and Perry Laboratories, Gaithersburg, MD) was added to each well and development was monitored by determining the optical density (OD) at 405 nm in an ELISA reader.

### Isolation and Analysis of Pulmonary CD4+ and CD8+ T cells

Cells were isolated and quantitated from the lungs as previously described [34]. Briefly, lungs were removed and ground between two frosted glass slides in PBS with 1% BSA and mononuclear cells were isolated from the homogenate on 40 to 70% Percoll gradients and enumerated on a Z2™ Coulter Counter^® ^(Beckman Coulter, Hialeah, FL). Labeling and flow cytometry analysis were performed as previously described [35]. The cells were labeled with an APC-conjugated Hamster anti-Mouse CD3 monoclonal antibody, a FITC-conjugated rat anti-mouse CD4(L3T4) monoclonal antibody, and a PE-conjugated rat anti-mouse CD4 (Ly-2) monoclonal antibody. All antibodies were obtained from BD Biosciences Pharmingen (San Diego, CA). Cells were analyzed on a FACSCalibur™ Flow Cytometry System (BD Biosciences, San Jose, CA).

### Statistical Analysis

Unpaired t tests were used to compare results of experiments between two groups. Multi-group comparisons between all groups in an experiment were performed by one-way analysis of variance (ANOVA) followed by the Tukey-Kramer test for multiple comparisons. All calculations were done with INSTAT (Graph Pad Software for Science, San Diego, CA). Significance was accepted when the P value was < 0.05 (2-sided).

## Results

### Environmental exposure to *P. murina *leads to the development of a transient colonization in immunocompetent KO mice

**Figure 1:**
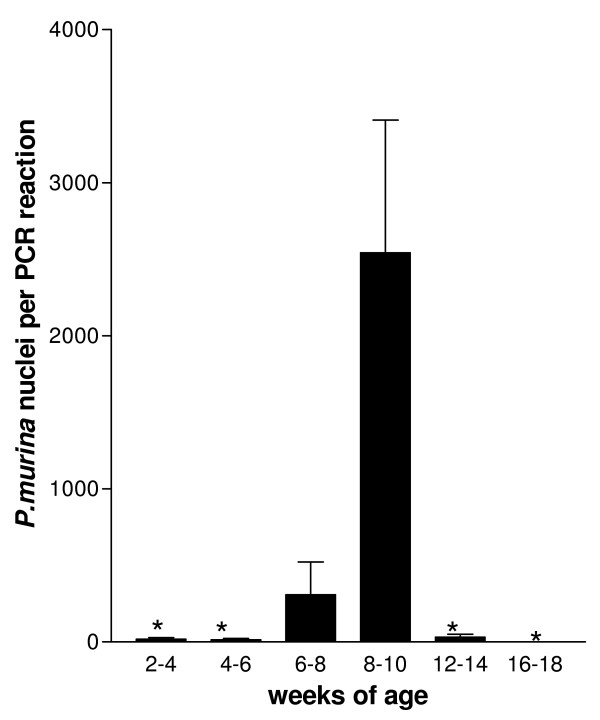
**Analysis of the development and clearance of *P. murina *colonization in immunocompetent SP-A deficient (KO) mice over time**. Immunocompetent KO mice between 2 and 18 weeks of age with no experimental exposure to *P. murina *were sacrificed, RNA extracted from the lungs and cDNA synthesized. *P. murina *large mitochondrial ribosomal RNA gene (mtLSU) mRNA levels were quantitated in the samples by qPCR. The data are the mean ± the SEM and are expressed as *P. murina *nuclei per reaction. *p < 0.05 vs. 8–10 week old group as determined by ANOVA. Each group contained at least 5 mice.

Immunocompetent WT and KO mice, between the ages of 2–18 weeks, with no experimental exposure to *P. murina *were examined for *P. murina *colonization by testing for the presence of mtLSU message by RT-qPCR. All mice used in these analyses were bred and housed under identical conditions as described in the material and methods. The mice were grouped at two-week age intervals and were only caged with mice within the group. Mice within a group were housed together up to 5 mice per cage. Some cages contained less than 5 mice depending on the number of mice in a group. All mice were housed in the same room. No *P. murina *specific mtLSU message was detected in WT mice of any age. In KO mice, the level of detection of the mtLSU message increased over time peaking in mice 8–10 weeks of age and then declined to undetectable in mice 16–18 weeks old (Fig 1). The percentage of mice within a group with detectable mtLSU also varied over time (Table 1). The frequency of the detection peaked in the 8–10 week group when mtLSU was detected in all of the mice Vimentin was used as a housekeeping gene marker to verify that the failure to detect mtLSU message in negative mice was not due to poor quality RNA or cDNA in the samples. Vimentin message was detected in all of the mice and no significant differences in the levels of expression were evident (data not shown). Lungs from immunocompetent KO and WT mice were also evaluated for the presence of *P. murina *cyst forms by CEV staining. The level of colonization in the KO mice was below the limit of microscopic detection as demonstrated by the inability to detect cyst or nuclei forms in the lungs of any of the mice following CEV or DQ staining by standard enumeration techniques. In this manuscript, the term colonization will be used to describe the presence of the low-level *P. murina *infection that appears to transiently exist in immunocompetent KO mice.

**Table 1:** Percentages of SP-A deficient mice with colonized with *P. murina *as determined by quantitative PCR detection of *P. murina *large subunit ribosomal RNA gene transcripts.

Weeks of Age	# infected mice	# of mice per group	% infected
2–4	6	14	42.9
4–6	4	16	25.0
6–8	12	18	66.7
8–10	12	12	100.0
12–14	4	10	40.0
16–18	0	5	0.0

### Immunosuppression induces heavy *P. murina *infection in KO mice following environmental exposure

**Figure 2:**
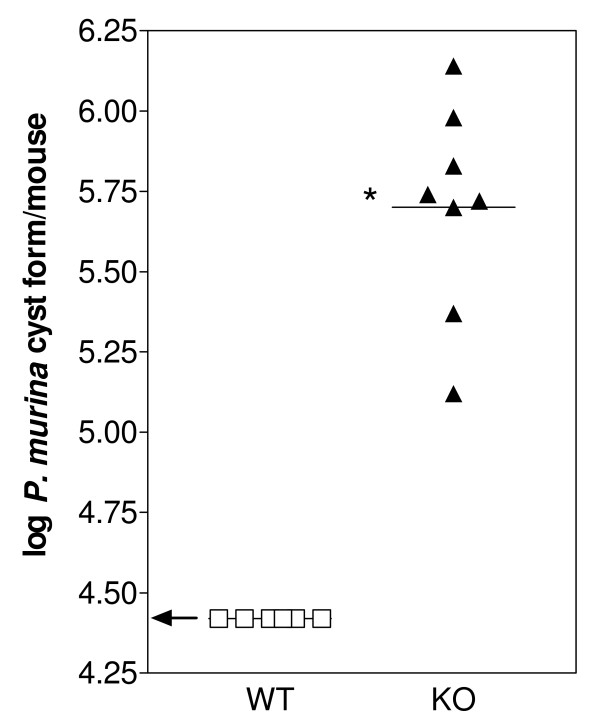
**Development of heavy *P. murina *infection in immunosuppressed Wild-Type (WT) and SP-A deficient (KO) mice**. WT (open square) and KO (black triangle) mice were immunosuppressed for 4 weeks, sacrificed and *P. murina *cysts forms in the lungs were quantitated microscopically. The limit of *P. murina *detection is approximately 2.5 × 10^4 ^cyst forms/lung (log_10 _4.4). Data were expressed as log_10 _cysts per mouse. Horizontal lines indicate mean log cysts per group. Each point represents a single mouse. *p < 0.01 as determined by t test. The arrow indicates the level of detection of microscopic enumeration.

C3H/HeN WT and KO mice with no experimental exposure to *P. murina *were immunosuppressed by the addition of dexamethasone to their drinking water for 4 weeks. The mice were sacrificed and lungs examined for *P. murina *infection by microscopic enumeration of cyst forms (Fig 2). *P. murina *infection only developed in the KO mice; no cyst forms were detected in WT mice whereas significant numbers of cyst forms were detected in KO mice. The development of active infection in the KO mice suggests that in the absence of SP-A, *P. murina *is able to survive in the immunocompetent host and initiate a heavy infection.

### Immunocompetent KO mice transmit *P. murina *infection to SCID Mice

This experiment was conducted to determine if immunocompetent KO mice colonized with *P. murina *were infectious. *SCID *mice were exposed to either immunosuppressed KO mice heavily infected with *P. murina *or immunocompetent KO mice colonized by *P. murina*. As a negative control, a third group of *SCID *mice was not exposed to KO mice. A fourth group of mice exposed to an immunocompetent WT mouse was not included in the experiment due to the availability of only a limited number of SCID mice

**Figure 3:**
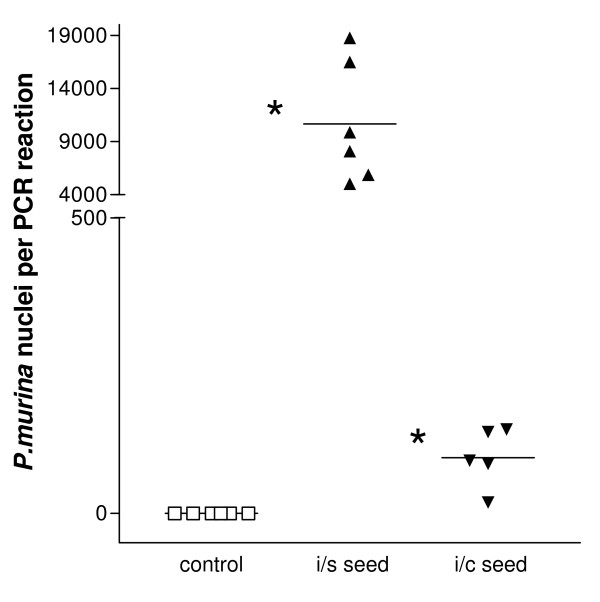
**Transmission of *P. murina *infection from immunocompetent SP-A deficient (KO) mice to severe combined immunodeficiency (*SCID*) mice**. *SCID *mice were housed in the same cage with immunosuppressed (i/s) (black triangles) or immunocompetent (i/c) (inverted black triangles) KO seed mice for two weeks. A third group of SCID mice was not exposed to any KO mice as a negative control (open squares). Transmission of Pneumocystis infection by direct exposure to an animal infected with Pneumocystis has been referred to as "seeding" and the infected mice are referred to as "seeds". The *SCID *mice were sacrificed, RNA extracted from the lungs and cDNA synthesized. *P. murina *large mitochondrial ribosomal RNA gene mRNA levels were quantitated in the samples by qPCR. The data are the mean ± the SEM and are expressed as *P. murina *nuclei per reaction. *p < 0.05 i/s seed vs. i/c seed by t test. Each group contained at least 5 mice.

Mice were sacrificed two weeks post exposure and examined for the presence of *P. murina *mtLSU in the lungs by qPCR. *P. murina *mtLSU message was detected in all of the mice exposed to immunosuppressed KO mice, in 5 of 6 mice exposed to immunocompetent KO mice, but not in any of the mice in the nonexposed negative control group (Fig 3). Higher levels of mtLSU were detected in mice exposed to the immunosuppressed KO mice than in mice exposed to immunocompetent KO mice. Similar vimentin message levels were detected in all of the mice (data not shown). The results do not demonstrate that immunocompetent WT mice are unable to transmit *P. murina *infection, but strongly suggest that immunocompetent KO mice with colonization may serve as a reservoir of *P. murina *infection and are capable of transmitting the infection to immunocompromised hosts.

### *P. murina *MSG specific serum antibodies detected in both immunocompetent KO and WT mice

To further examine the development of the humoral response, serial serum samples were obtained from individual KO and WT mice by retro orbital eye bleed at 4, 6, and 8 weeks of age. At 10 weeks of age, the mice were sacrificed and a terminal blood sample was collected by cardiac puncture. This procedure allowed us to monitor the development of *P. murina *specific antibodies over time in the same mouse. The samples were tested for the presence of *P. murina *specific antibodies by ELISA, using a recombinant fragment of *P. murina *MSG spanning amino acids 336–437.

**Figure 4:**
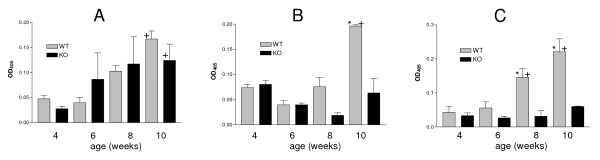
**Analysis of the *P. murina *serum antibody response in immunocompetent Wild-Type (WT) and SP-A deficient (KO) mice**. *P. murina *MSG specific IgG1 (**A**), IgG2a (**B**) and IgG2b (**C**) serum antibodies in immunocompetent WT and KO mice with no experimental exposure to *P. murina *were analyzed by ELISA in mice 4, 6, 8, and 10 weeks of age. Each group contained 4 mice. Data are expressed as the Mean ± the SEM of the OD_405 _for each group *p < 0.01 KO vs. WT at time point, +p < 0.01 increase over time within a group, as determined by ANOVA.

MSG-specific IgG1 antibodies levels increased over time in both the WT and KO mice and no significant differences were detected between the two groups at any age (Fig 4A). Significant differences in the levels of MSG specific IgG2a and IgG2b were detected between WT and KO mice in older mice. WT mice had more MSG-specific IgG2a than KO mice at 10 weeks of age (Fig 4B) and IgG2b levels were higher in 8 and 10 week old WT mice (Fig 4C). IgG2a and IgG2b MSG-specific antibodies also increased significantly over time in the WT mice but not in the KO mice (Fig 4A, B and 4C). These results demonstrate that both WT and KO mice mount a humoral immune response to *P. murina*. However, it is not clear if the antibodies play a role in protecting the WT mice from colonization.

### Immunocompetent KO mice display an enhanced cellular pulmonary immune response

**Figure 5:**
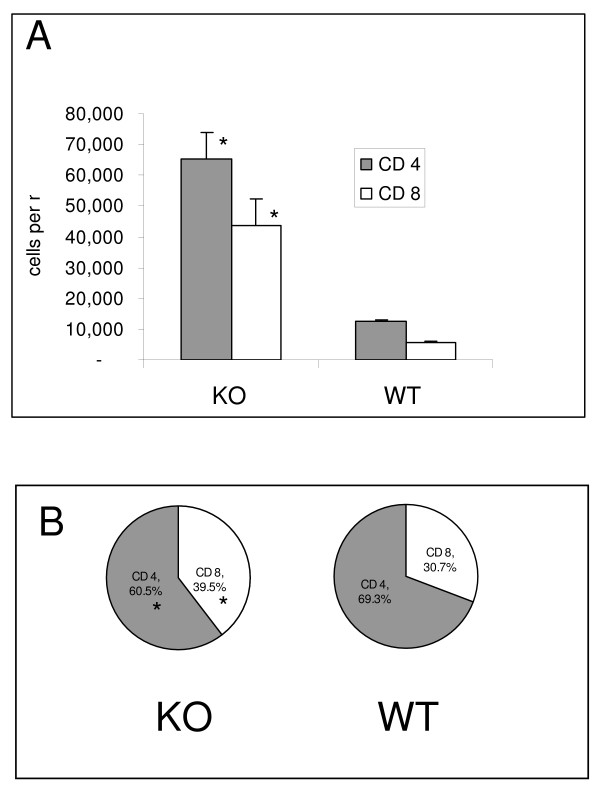
**Analysis of the cellular pulmonary immune response in immunocompetent SP-A deficient (KO) and Wild-Type (WT) mice**. Mononuclear cells were isolated from the lungs of KO and WT mice with no experimental exposure to *P. murina*. CD4+ and CD8+ T cell populations were identified by cell surface antibody labeling and quantitated by flow cytometry. **A**. Mean ± the SEM of the number cells per mouse. **B**. Mean percentage of CD4+ and CD8+ T cells per mouse. There were 10 mice in the KO group and 8 mice in the WT group. * p < 0.01 KO vs. WT as determined by t test.

These analyses were performed to determine if the *P. murina *colonization in the KO mice stimulates a cellular pulmonary host response. Mice 6–8 weeks of age were chosen for these analyses because it was predicted that the pulmonary cellular response may precede the peak of *P. murina *colonization. Mononuclear cells were isolated from the lungs of KO and WT 6–8 week old immunocompetent mice and CD4+ and CD8+ T cell populations were enumerated by flow cytometry (Fig 5). Significantly more CD4+ and CD8+ T cells were isolated from the lungs of KO mice than from WT mice and the CD4+:CD8+ T-cell ratio was significantly lower in the KO mice (Fig 5A). The ratio in the KO mice was approximately 1.5 and in the WT mice it was 2.2. The reason behind the alteration of the CD4+:CD8+ T-cell ratio is reflected by the comparison of the percentages of these cell types in KO and WT mice (Fig 5B). A significantly lower percentage of CD4+ T-cells and a corresponding significantly higher percentage of CD8+ T-cells were found in the KO mice than in the WT mice.

### CD4+ T cell depletion inhibits clearance of *P. murina *from the lungs of KO mice

**Figure 6:**
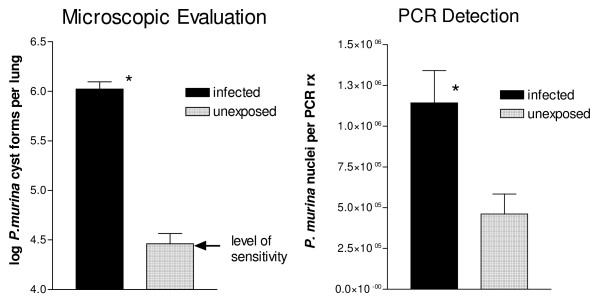
**CD4+ T cell depletion inhibits clearance of *P. murina *from the lungs of SP-A deficient (KO) mice**. KO mice infected with *P. murina *by intratracheal inoculation or unexposed KO mice were treated with a CD4 depleting antibody for four weeks. The animals were sacrificed and *P. murina *infection was evaluated by microscopic enumeration of cyst forms or by quantitation of the *P. murina *large mitochondrial ribosomal RNA gene by qPCR. The cyst form data are expressed as log_10 _cyst forms per lung. The level of sensitivity in this assay is 4.4 log_10 _cyst forms per lung. The qPCR data are the mean ± the SEM and are expressed as *P. murina *nuclei per reaction. *p < 0.05 as determined by t test.

The previous results demonstrate that KO mice harbor higher levels of CD4+ T-cells in their lungs. To determine if CD4+ T-cell depletion inhibits clearance of the colonization, unexposed KO mice and KO mice infected with *P. murina *by intratracheal inoculation, were depleted of CD4+ T-cells by treatment with Gk1.5 antibody. After 4 weeks, mice were sacrificed and the level of infection was evaluated by microscopic enumeration and by qPCR. *P. murina *cyst forms were detected in only 4 out of 10 unexposed KO mice whereas 15 out of 15 KO mice infected by intratracheal inoculation developed detectable levels of *P. murina *cysts forms (Table 2). In addition, there were significantly fewer cyst forms in the unexposed mice that had detectable levels of organisms (Fig 6A). The lungs were also analyzed for the presence of *P. murina *by qPCR detection of mtLSU expression (Fig 6B). *P. murina *was detected by this assay in all of the unexposed mice as well as in all of the mice infected by intratracheal inoculation (Table 2). Significantly higher levels of mtLSU expression were detected in the inoculated mice compared to the unexposed mice. The level of infection present in the unexposed KO mice was higher than levels previously detected in the immunocompetent KO mice.

**Table 2:** Percentages of unexposed and inoculated SP-A deficient mice with detectable levels of *P. murina *following CD4 cell depletion.

		Evaluation of Infection			
		
		# infected mice		% infected	
	
Group	# of mice per group	microscopic	RT-qPCR	microscopic	RT-qPCR
Inoculated	15	15	15	100	100
Unexposed	10	4	10	40	100

## Discussion

The results of these studies indicate that immunocompetent SP-A KO mice, but not WT mice, harbor viable and actively replicating *P. murina *following environmental exposure. The source of *Pneumocystis spp*. infection in humans and animals remains unknown. Various species have been detected by environmental sampling techniques, suggesting a possible environmental source of the infection; yet, the infectious potential of these environmental samples has not been demonstrated [36-39]. Human-to-human transmission of *P. jirovecii *has been identified by epidemiology studies that identified direct transmission and clusters of infections [40-42]. Conversely, other studies determined that person-to-person transmission of *P. jirovecii *did not appear to contribute significantly to the spread of the disease [43,44]. Several studies have demonstrated that recurrence of PcP following successful treatment involves *P. jirovecii *of a different strain from the initial infection, suggesting that the recurrence is the result of infection from an exogenous source that may be either due to environmental exposure or person-to-person transmission [45,46].

The development of sensitive PCR techniques led to the search for the presence of low levels *P. jirovecii *in the lungs of immunocompetent and immunocompromised humans [47]. It had long been thought Pneumocystis remained in a latent stage within the lungs of immunocompetent individuals and became reactivated during immunosuppression; however, results from early studies indicated that *P. jirovecii *was not present in lungs of non-immunosuppressed individuals [48-50]. In addition, a more recent study also indicated that healthy subjects may not be colonized with *P. jirovecii *[51]. These results suggest that *P. jirovecii *does not exist in a latent stage in immunocompetent individuals.

Although, *P. jirovecii *colonization was not detected in healthy individuals, *P. jirovecii *colonization has been described in some hospitalized patients with moderate to severe underlying immunodeficiency suggesting that this patient population could serve as a reservoir of infection [52]. Results from a recent study suggest that *P. jirovecii *colonization in HIV + hospitalized patients may also be a source of infection. In that study, it was found that 68% of hospitalized HIV + patients with non PcP pneumonia were colonized with *P. jirovecii *[53]. In another recent study, *P. jirovecii *colonization was detected in 46% of the autopsy specimens of HIV+ individuals [7]. Also, in that study, cigarette smoking and city of residence were discovered to be risk factors for colonization. In a study, examining bronchoalveolar specimens, a lower level of colonization was detected in persons infected with HIV, but these authors also propose that this carriage may be a reservoir of infection [8]. *P jirovecii *colonization in patients with other lung diseases such as cystic fibrosis and chronic obstruction pulmonary disease, without significant underlying immunosuppression, has also been described [54].

The findings of an early study indicated that *P. jirovecii *colonization was not associated with cystic fibrosis. [55]. However, in two more recent studies colonization rates of 7.4% and 21.5% were detected in individuals with cystic fibrosis [56,57]. Interestingly, it has also been show that *P. jirovecii *colonization in cystic fibrosis patients is a dynamic process, in which clearance and recolonization occurs over time [58].

Several reports of *P. jirovecii *colonization in patients with chronic obstructive pulmonary disease (COPD) have been published. In an early study, a colonization rate of only 10% was detected and the authors postulated that since this rate of colonization was similar to the rate of PcP being seen in immunocompromised patients at their site, PcP arose from a latent infection and was not the result of a new infection [59]. *P. jirovecii *colonization has also been linked with severity of COPD as measured by spirometric lung function in a study that examined autopsy specimens. In that study, colonization was detected in 37% of smokers with severe COPD, but only 5.3% of those with less severe disease [11]. *P. jirovecii *colonization in persons with COPD has also been associated with higher levels proinflammatory cytokines, suggesting that the host mounts an immune response to the colonization [12]. In this study, colonization was detected in 55% of induced sputum samples from COPD patients, a much higher rate of colonization than previously reported. The variation in reported colonization rates may be related to the type of samples that were tested or due to differences in the detection methods that were used. Alternatively, the different colonization rates may indicate that some demographic areas may have higher rates of colonization than others, as was reported in colonization rates in HIV+ individuals [7].

Animal-to-animal spread of the infection has been well documented and supports the role of immunocompetent hosts as reservoirs of *Pneumocystis spp*. infection. In studies using mouse models, transmission of *P. murina *infection through cohousing with infected mice initiates infection in both immunocompetent and immunocompromised animals [60,61]. In normal mice, the colonization is self-limiting; immune-mediated clearance occurs 5–6 weeks post initiation of infection and involves both humoral and cellular immune responses [5,62]. Normal mice with this transient colonization have also been shown to be able to transmit the infection, further supporting the normal host as a reservoir of infectious organisms [60,62].

It was surprising to detect environmental exposure of *P. murina *within our barrier animal facility; however, this finding may not be totally unexpected. Previously, both *P. murina *and *P. carinii *outbreaks have been described in commercial animal facilities, demonstrating the ability of *Pneumocystis *to circumvent isolation systems designed to limit cage-to-cage exposure. It has been shown that air may circulate into cages with micro isolator tops not through the filter on top of the cage, but rather through the edges of the cage which may not be rigorously sealed [63]. Even though cages are changed within a laminar flow hood, another source of potential environmental exposure to *P. murina *would be during cage changing procedures. Cages of mice used in these studies were changed 2–3 times per week which may have lead to increased environmental exposure and development of colonization in the KO mice. This could result in cross-contamination of cages within the colony. Colonization is a very common finding in immunocompetent rat colonies that have been screened by PCR, but it usually goes undetected [64]. A systematic survey of normal mouse colonies has not been performed, but both colonization and active *P. murina *infection in several genetically immunodeficient mouse colonies has been described [65,66]. These findings demonstrate that colonization of laboratory animals occurs in spite of maintenance of strict barrier conditions, and suggests that these practices are not sufficient to prevent the development of *Pneumocystis *colonization [66,67].

In the present report, both WT and KO mice apparently encountered *P. murina *through environmental exposure within the animal facility, based on the development of *P. murina *specific serum antibodies in both strains of mice. In WT mice, it seems that the *P. murina *is eliminated from the lungs and never establishes an infection; yet, in KO mice the *P. murina *establishes a transient colonization. Based on these results, we propose that there are two potential outcomes of environmental exposure to *Pneumocystis *in the immunocompetent host: 1) *Development of transient colonization*. In this scenario, *Pneumocystis *escapes detection by the innate immune response and establishes a colonization that may be eventually cleared by the adaptive immune response. 2) *Elimination of Pneumocystis without development of colonization*. In this situation, the innate immune response recognizes and eliminates the *Pneumocystis *prior to it being able to establish an active infection.

It is likely that the level of environmental exposure within the animal facility is very low and sporadic. WT mice appear to be able to defend against such a low level exposure; however, the absence of SP-A in the KO allows the *P. murina *to escape innate host responses, which are likely to be involved in protection of the host from initial infection. It is interesting to note that at the time that these studies were performed at the UC LAM facility, colonization was not detected in *SCID *mice housed under the same conditions in microisolator cages. It was thought that the absence of colonization in the *SCID *mice reflected the presence of SP-A and/or residual immune function. Recently, our mouse colonies have been relocated to a different animal facility. The housing conditions in this facility are similar to conditions used in the previous facility, but sporadic colonization has been observed in *SCID *mice housed in microisolator cages in this facility. These new findings indicate that *SCID *mice are susceptible to development of colonization following environmental exposure, and support the idea that all of the mice within the facility are environmentally exposed to *P. murina*. These data also emphasize the complexities of developing animal models of *Pneumocystis *colonization. On the one hand, acquisition of *P. murina *colonization via natural environmental exposure most closely mimics the situation in humans, as other methods of transmitting *P. murina *such as intratracheal inoculation, intranasal inoculation, or co-housing infected with uninfected mice ("seeding") involve larger or more intense types of exposure. On the other hand, environmental exposure studies are uncontrolled and become difficult to interpret when *P. murina *infection already exists in some members of an animal colony.

Although immunocompetent SP-A KO mice are susceptible to *P. murina *colonization, they can limit and ultimately clear the infection. Not surprisingly, CD4+ T cells appear to play an important role in this process. Increased numbers of CD4+ T-cells were detected in the KO mice compared to the WT mice, and antibody mediated CD4+ T-cells depletion resulted in higher levels of infection in the KO mice. Further characterization of the SP-A independent factors involved in clearance of colonization will provide insight into the mechanisms immunocompetent hosts utilize to defend against *Pneumocystis *infection.

SP-A is a member of the collectin family of proteins, innate immune molecules containing a collagen-like amino terminus and a C-type lectin carbohydrate recognition domain at the carboxyl terminus [15]. Structurally similar to C1q, mannose-binding protein and surfactant protein D (SP-D), it is secreted by type II alveolar epithelial cells. SP-A KO mice have provided valuable information on the role of SP-A in host defenses against *P. murina *and other organisms [17]. These animals have normal lung morphology, physiology, surfactant phospholipids and other surfactant proteins (SP-B, SP-C or SP-D), but are deficient in tubular myelin [16]. KO mice have impaired clearance of infectious agents following experimental exposure, which is usually accompanied by increased proinflammatory cytokines [17]. A recent study demonstrated that SP-A null mice raised in an environment heavily contaminated with bacteria were more susceptible to bacterial peritonitis than WT mice [68]. Although the lungs were not involved in the infection in this model, the results demonstrate that SP-A KO mice may also be susceptible to infections following environmental exposure.

It has been previously shown that immunosuppressed SP-A KO mice are more susceptible to *P. murina *infection, and when immunosuppressed, they develop higher levels of PcP than WT mice [24,25]. Immunocompetent KO mice also display delayed clearance following infection by intratracheal inoculation compared to WT mice [69]. However, SP-A did not enhance clearance of *P. murina *in a steroid treated mouse model of infection. In this model, KO mice cleared the infection just as efficiently as WT mice following withdrawal of the corticosteroid induced immunosuppression [34].

SP-A plays a role in early host defenses by enhancing the uptake of infectious agents by alveolar macrophages and has been shown to facilitate the adherence and/or phagocytosis of most of the organisms with which it has been tested[70]. In vitro studies have demonstrated that under certain conditions SP-A enhances attachment of *Pneumocystis *to AMs; however, inhibition of *Pneumocystis *binding to alveolar macrophages by SP-A has also been reported [71,72]. In our laboratory, preincubation of *P. murina*, which was isolated from KO mice, with SP-A resulted in enhanced attachment to mouse alveolar macrophages [24].

SP-A also displays other protective activities in the host response. Binding of SP-A to gram-negative bacteria, *Mycoplasma pneumoniae*, and *Histoplasma capsulatum *has been shown to result in killing of the organisms [23]. SP-A also increases aggregation of organisms that leads to enhanced clearance from the respiratory tract by mucociliary actions [17]. In addition to these direct activities, SP-A interacts with a variety of immune cells and modulates their responses to pulmonary pathogens [15]. SP-A has been shown to inhibit maturation of dendritic cells and limit their ability to activate T cells [73]. In addition to its regulation of the innate immune response, there is rising interest in the interaction of SP-A with the adaptive immune system [74]. A recent report showed that SP-A binds to the Fc portion of IgG and facilitates the uptake of IgG coated erythrocytes by AMs [75]. These other SP-A activities have not been characterized in the host response to *Pneumocystis*; however, it is likely that prevention of colonization involves both innate and adaptive immune response mechanisms and that SP-A may have additional activities beyond enhancing uptake by alveolar macrophages.

The current study demonstrates a new role for SP-A in protecting the immunocompetent host from *P. murina *infection by preventing the normal host from becoming infected with *P. murina *following environmental exposure. The protective effects of SP-A in this situation, may involve direct action of the molecule on *P. murina *viability, indirect modulation of other factors of the host immune response, or enhanced recognition of *P. murina *by alveolar macrophages [23,23,72,76]. SP-A could also exert its protective effects by a combination of all these activities.

*Pneumocystis *colonization in immunocompetent hosts may be a mechanism that the organism uses as a survival and transmission strategy, thus is relevant to the care of immunocompromised patients. The detection of significant *P. jirovecii *colonization rates in humans suggests the need for caution in interactions between hospital staff and immunocompromised patients [77]. Identification of factors involved in protecting the immunocompetent host from becoming infected with *Pneumocystis*, may provide tools to eliminate this reservoir of infection and reduce spread of the disease to highly susceptible immunocompromised patients.

A role for SP-A in host defense against *P. murina *infection has been clearly demonstrated in KO mouse models and by in vitro experiments [24,26]. However, the role of SP-A in the host response to *P. jirovecii *infection in humans has not yet been established. It has been shown that immunodeficient individuals with HIV/AIDS and PcP have increased levels of SP-A in BALF compared to individuals with bacterial pneumonias [78]. In that work, the authors did not demonstrate a functional role for SP-A in the host response to *P. jirovecii *in humans, but the results suggest that SP-A may be involved. The results presented in the current study indicate that SP-A may also be involved in protecting the immunocompetent host from *Pneumocystis *colonization. As described above, the pathophysiologic mechanisms associated with *P. jirovecii *colonization in humans, in addition to immunodeficiency, include chronic pulmonary diseases and smoking [79]. In humans, SP-A levels within BALF have been show to vary considerably among normal individuals and also among persons with various lung diseases [80]. It is interesting to note that SP-A levels have been shown to be decreased by smoking and smoking has also been identified as a risk factor for *P. jirov*ecii colonization. Recent work has also demonstrated that exposure to cigarette smoke leads to increased burdens of *P. murina *in mice [81]. Two unique variants of SP-A (SP-A1 and SP-A2) are expressed from two different genes in humans and more than 30 alleles have been described for the SP-A genes [82]. It has been shown that the SP-A variants have functional differences and postulated that SP-A2 may be more active in the innate immune response than SP-A1 [83,84]. Based on these findings it is possible that decreased SP-A levels or expression of particular SP-A variants may lead to enhanced susceptibility to *P. jirovecii *colonization in humans.

## Conclusion

The data in this study support an important role for SP-A in protecting the immunocompetent host from *P. murina *colonization, and provide a model to study *Pneumocystis *colonization acquired via environmental exposure in humans. The results also illustrate the difficulties in keeping mice from exposure to *P. murina *even when housed under barrier conditions.

## Abbreviations

PcP: *Pneumocystis *pneumonia; UC: University of Cincinnati; LAM: Laboratory Animal Medicine; SP-A: surfactant protein A; WT: wild type; KO-SP-A: knockout; qPCR: reverse-transcriptase quantitative PCR; ELISA: enzyme-linked immunosorbent assay; mtLSU: large mitochondrial ribosomal RNA gene; SCID: severe combined immunodeficiency.

## Competing interests

None of the authors has a commercial or other association that might pose a conflict of interest.

## Authors' contributions

MJL led he design and supervision of the experiments, data analysis, and preparation of the manuscript. ADA carried out the animal studies and ELISA. JAD carried out the molecular studies. PDW provide overall leadership to the design of the experiments, data analysis, and preparation of the manuscript.
